# The impact of the anthropause caused by the COVID-19 pandemic on beach debris accumulation in Maui, Hawaiʻi

**DOI:** 10.1038/s41598-023-44944-4

**Published:** 2023-10-18

**Authors:** Jens J. Currie, Florence A. Sullivan, Elizabeth Beato, Abigail F. Machernis, Grace L. Olson, Stephanie H. Stack

**Affiliations:** 1 Pacific Whale Foundation, Wailuku, HI 96793 USA; 2 Pacific Whale Foundation Australia, Urangan, QLD 4655 Australia

**Keywords:** Environmental sciences, Environmental impact

## Abstract

The COVID-19 pandemic and subsequent travel restrictions led to a considerable reduction in tourism and human activity on Maui, presenting a unique opportunity to study debris accumulation on local beaches during changing levels of human activities. Standardized daily debris accumulation surveys were completed at two beach sites in Maui, Hawai ‘i before (2017) as well as throughout the initial year of the pandemic (2020–2021) and allowed for the assessment of pandemic-related restrictions on marine debris accumulation trends. Throughout the pandemic, reduced beach use due to higher lockdown levels had significant impacts on debris accumulation at both sites, but only one of the two sites experienced a significant decrease (~ 90% reduction) in debris accumulation rates when compared to the same months in 2017. Daily accumulation rates across two sites increased from an average of 16 items/100 m during peak lockdown levels to 43 items/100 m when restrictions eased. The observed fluctuations in debris accumulation rates, driven by changes in tourism and travel restrictions during the COVID-19 pandemic emphasize the importance of proactive measures to protect the natural environment, including source reduction and effective legislation for waste prevention. By addressing both local and remote sources of debris and focusing on reducing waste at its source, it is possible to mitigate the impacts of debris accumulation on coastal environments and marine life in Hawaiʻi.

## Introduction

Plastic production and its environmental consequences have evolved into a global concern of paramount importance^1^. The widespread distribution of plastic waste on beaches, nearshore waters, and the open ocean poses significant threats to marine life^2^, ecosystems^3^, and local economies^1^. Beach debris originates from a diverse array of sources, including discarded/forgotten items from beachgoers, marine debris washed ashore, and storm water/river outflows carrying urban pollutants into coastal areas^4–6^. Some of the most polluted beaches can be found in remote uninhabited regions^7–10^, highlighting the global reach and persistence of marine debris that can wash ashore, unrelated to beach use. In Hawai‘i, debris regularly accumulates on beaches^11–13^, and in some areas in excess of 1000 items/100 m/day^14^, which is attributed to the proximity of the convergence zone north of the Hawaiian Islands^8^. Beach debris gathered from Maui’s beaches on the island's leeward and heavily-resorted side, consists of 85–90% land-based items^14^. Debris accumulation has been linked to tourism and tourism mindset^15^, both on Maui and elsewhere in the world, with areas that experience high tourism having higher proportions of debris^16,17^.

The global COVID-19 pandemic^18^ spurred nations across the world to enforce travel bans and quarantines^19–21^, creating an opportunity to evaluate different sources of marine debris generation. By the end of March 2020, all countries had introduced some form of travel restrictions, with 27% of countries completely shutting their border to international travelers^22^. Reduced human activity resulting from COVID-19 travel restrictions has already been shown to lead to reductions in human impacts elsewhere and has been termed the “anthropause” to signify the “considerable global slowing of modern human activities”^23^. In South Africa, street litter loads decreased roughly by a factor of three during the strict lockdown levels^24^. In Ecuador and Europe, local surveys suggested that beaches had significantly less marine debris and improved ecological conditions over the course of lockdowns^25^, with this trend linked to reduced tourism activities in some areas^26^. However, some areas reported increased impact, with Personal Protective Equipment, such as facemasks, becoming more prevalent at beaches throughout various regions^27–29^.

The state of Hawai‘i began implementing a mandatory 14-day quarantine for all travelers originating outside of Hawai‘i on March 26, 2020^30^ resulting in a drastic reduction in tourism arrivals^10,31^. The 14-day quarantine was then extended on April 1, 2020 to also include all interisland travel and the state saw monthly visitor counts drop to 4,564, down from 856,250 during the same period in 2019^30^. Maui experienced similar declines, with 647 visitors on Maui for all of April in 2020 versus 248,042 visitors the year prior^30^. In addition to the travel quarantine, residents were advised to stay at home, with state and county beaches closed except for essential activities such as fishing and exercise^30^. In late April, restrictions started to ease with the lifting of the stay-at-home-order and beaches were reopened with social distancing requirements in June 2020^30^. However, when compared to the previous year, the restrictions brought on by COVID-19 still impacted tourism on Maui, with arrivals reduced by 98% in August^31^. Mid-October 2020, the state provided a pre-travel program, which allowed visitors from the continental USA to bypass the 14-day quarantine requirement, resulting in 63,740 visitors on Maui for the month of November^31^. The lockdown measures and travel restrictions of the COVID-19 pandemic caused the number of tourists arriving in Maui to decrease significantly, as well as the number of beach users^31,32^.

On Maui, tourism is one of the primary drivers of the local economy^33^, with ~ 9.8 million visitors arriving each year^31^. The high frequency of ocean and beach-based activities, favored by both tourists and residents alike, results in beach litter through improperly disposed products, especially single-use items^34^. Previous research on Maui has found that the beaches on the leeward side of the island received the highest proportion of debris from land-based sources (i.e., originating from Maui^14^). The variations of land-based debris accumulation in Maui was attributed to seasonal beach use with high visitor days corresponding with high debris loads^17^.

The anthropause caused by the COVID-19 pandemic provided a unique opportunity to assess debris accumulation with changing levels of human activities on local Maui beaches throughout the course of the pandemic. This paper aimed to compare daily debris accumulation rates at two beaches (1) during the COVID-19 pandemic as lockdown restrictions eased and tourism numbers and beach use increased; (2) before and during the COVID-19 pandemic.

## Methods

### Study area

**Figure 1:**
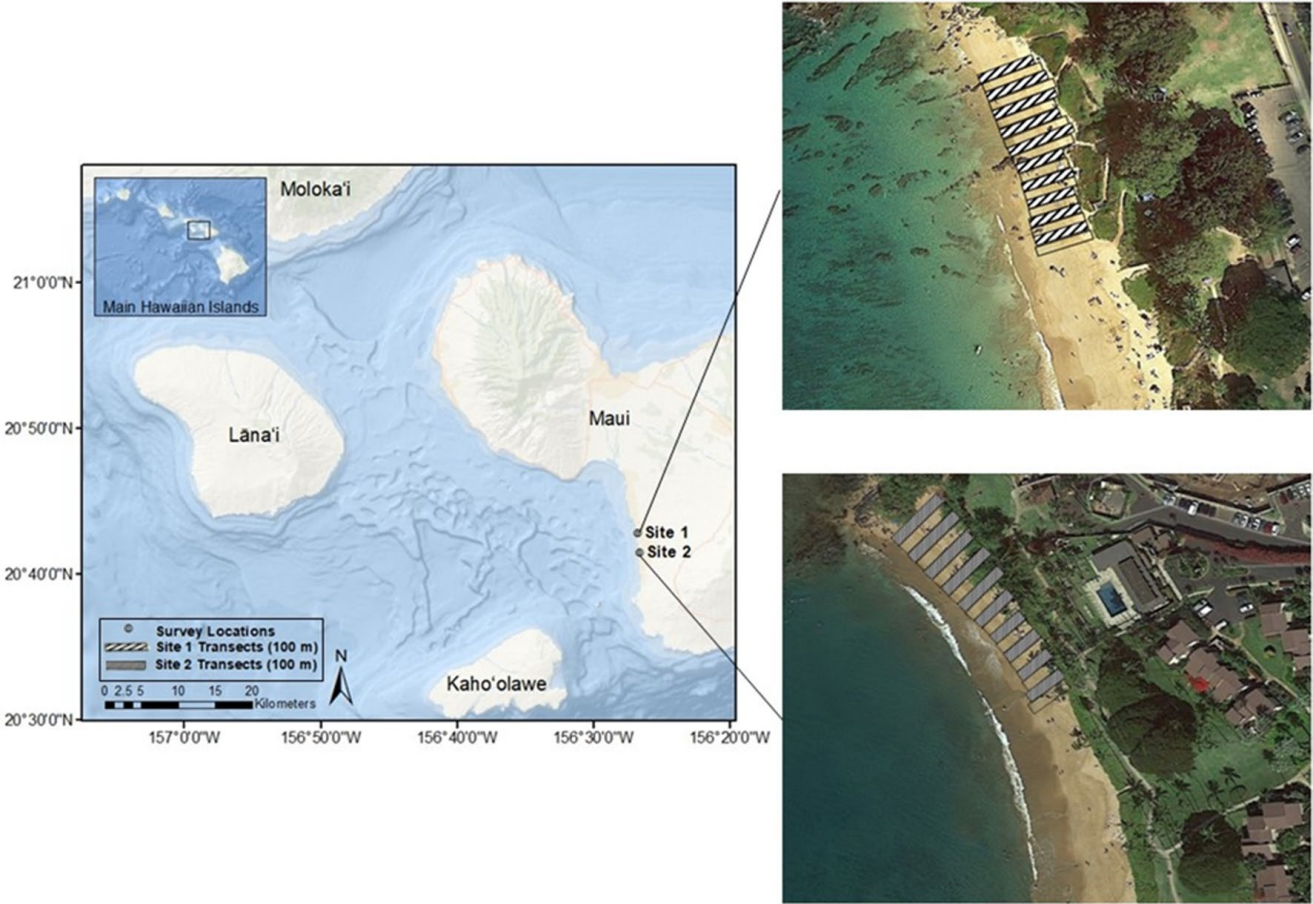
Map showing the location of the two survey sites on Maui with inserts showing the topographic details and locations of the 100 m transects. Site 1: Kama‘ole Beach Park III; Site 2: Ulua Beach. *Ocean Base Map Source: Esri,GEBCO, NOAA, National Geographic, DeLorme, HERE, Geonames.org, and other contributors*^35^*.*

As a result of the COVID-19 pandemic, the state of Hawai‘i began lockdown and quarantine measures in March of 2020, restricting most activities in Maui to essential services only. Marine debris surveys were initiated in May 2020, after beach access was opened for essential activities, including research. To allow for comparisons to pre-pandemic debris accumulation trends, two sites of similar size that were previously surveyed in 2017 were selected (Fig. 1). Site 1 (Kama‘ole Beach Park III; 20.71389, -156.44661) is a Maui County beach park containing a large grassy area adjacent to a sandy beach and is equipped with picnic tables, grills, restrooms, and outdoor showers. It is easily accessed by several condominium and housing developments and used by both tourists and residents. Site 2 (Ulua Beach; 20.69159, -156.44429) is a non-County beach with a small grassy area, restrooms, and outdoor showers behind the sandy beach. Site 2 is located near a large resort as well as large private residences and is used by both tourists and residents. Both sites are bounded by rocky outcroppings on the leeward shore of Maui, Hawai‘i.

### Data collection

Surveys were conducted once per week following the accumulation survey protocol outlined in the NOAA Marine Debris Shoreline Survey Field Guide^36^. Research surveys were carried out by trained research staff and the survey on day one was to remove all debris from the beach and allow for a standardized 24-hour (hr) accumulation period for the survey on day two. Daily accumulation surveys were employed to mitigate underestimation of debris accumulation arising from the re-deposition of beach debris into the ocean, as previously observed in Maui^14^. Furthermore, this approach reduced the likelihood of impromptu cleanup efforts by beachgoers, a scenario that might have been more prevalent following the relaxation of lockdown restrictions, as their window for debris removal at the survey location was limited to a 24-h timeframe. However, it remains important to acknowledge the inability to fully account for these factors and their potential impact on our accumulation rate calculations cannot be ruled out. Two surveys (cleaning and accumulation) were conducted weekly for one year from May 2020—May 2021 and used the same methodology employed from July to December in 2017. A 100 m (m) transect was randomly placed on each beach in Arc Map 10^37^ to determine the start and end positions of the survey area (see inserts in Fig. 1). To ensure sampling consistency, a handheld GPS was used to find the same start and end survey areas throughout the study period. Each survey area was divided into 20 five-meter-wide strips running perpendicular to the shoreline. The transects were traversed by the trained research staff at the same time each day who moved from the waterline to the back of the shoreline, indicated by a vegetation barrier, until the entire survey area was covered. During the survey, any piece of debris ≥ 1 centimeters (cm) was removed and documented. Limiting the count to debris items of this size or larger effectively reduced the likelihood of tallying beach debris that had been previously buried and later exposed due to foot traffic, which is more common for smaller debris items. It is essential to acknowledge that the study did not address the potential confounding effect of increased foot traffic, leading to ongoing exposure of more debris throughout the study, particularly as lockdown restrictions eased and foot traffic increased.

### Data processing

Daily debris counts (i.e. accumulation over 24 hr) were calculated by summing the total number of all debris items collected within the 100 meter (m) transect during the second survey day for each site and were used in all subsequent analysis.

### Data sources

Environmental indices of relative exposure index (REI)^38^, relative tidal range (RTR)^39^, and intertidal area (IA)^40^, for each site were calculated following previously published methods^11^ for the 24 hrs preceding the survey so that the indices represent the conditions under which the debris accumulated. Three beach profiles for each site were determined using methods in^41^ and were averaged to create a representative slope for each beach. Wind data (speed in kilometers (km)/hr and cardinal direction) were downloaded from public weather stations^42^ using a public weather scraper created by Karl Niebuhr^43^. The closest weather station for the Site 1 was KIHKIHEI53 located 0.8 km from the survey area, while the KHIKIHEI4 station was closest to Site 2 at a distance of 1.3 km. Tide heights (cm) at High/Low intervals were downloaded from NOAA Tides and Currents data clearinghouse^44^. Tides were taken from NOAA Makena station 1,615,202 as the closest representative for both survey sites located at 6.3 km and 3.8 km, respectively. Wave heights, recorded in meters at 30-minute intervals, were downloaded from the National Data Buoy Center ^45^. Station 51,213 was used as the closest representative for both survey sites located at 58.1 km and 58.5 km, respectively.

Beach users varied over time, and logistical constraints did not allow for accurate total counts for the 24 hrs preceding each survey. Since beach use is known to fluctuate with tourism numbers^11,46^, the count of daily domestic passenger arrivals was determined to be an appropriate proxy for tourism and beach use. Daily domestic passenger arrivals obtained from the Department of Agriculture and Department of Transportation included returning residents, intended residents and visitors. Counts did not include passengers on inter-island flights or flights from Canada as this information was not available. Data were downloaded from the State of Hawaiʻi’s Department of Business, Economic Development and Tourism webpage^47^.

**Figure 2:**
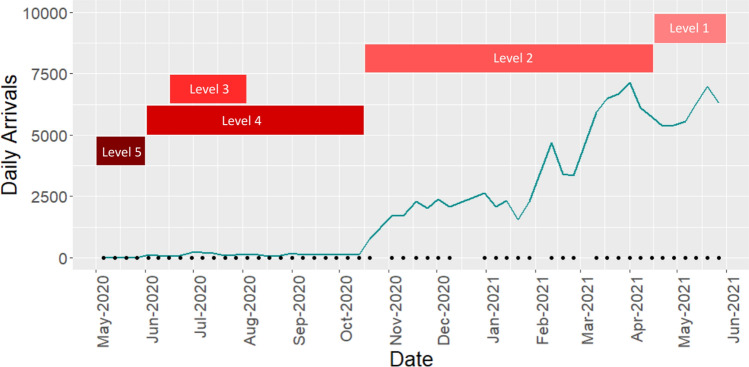
Daily visitor arrivals to Maui^31^ averaged weekly during the survey, accompanied by corresponding lockdown levels^30,32^, from the most restrictive (Level 5) to the least restrictive (Level 1). Each point on the x-axis denotes a weekly sampling date.

To establish a timeline of local restrictions relating to COVID-19, both the State of Hawai‘i^30^ and County of Maui emergency proclamations^32^ were reviewed to determine and record exact start and end dates of state and county mandates during the survey. Five levels were used to represent different restrictions that would likely impact the level of beach use in Maui County during the study period, with level 1 representing the lowest lockdown requirement and 5 the highest. The levels considered beach access, travel restrictions, face-covering mandates, and restaurant closures (Fig. 2; Table 1 Supplemental Materials).

### Data analysis

All calculations and statistical analyses were completed using R v. 4.2.0^48^. Significance levels were evaluated at a p-value of 0.05, with a value < 0.05 considered to be statistically significant.

### Changes in debris accumulation rates throughout the COVID-19 restrictions

A Generalized Additive Model (GAM) in the *mgcv* package^49,50^ was used to assess how the daily debris count changed as lockdown levels varied and visitor numbers fluctuated. Debris count was modelled as a function of both lockdown level and date (Julian day) separately for each site. Three additional environmental covariates, REI, RTR, and IA, were included in all models as they are known to impact coastal debris accumulation rates^14^. Although not included in final models, daily domestic arrivals, a proxy for tourism, was also included in initial models. All models were fitted using penalized regression splines with default smoothing values (10 knots) for each spline, and the smoothing parameters were estimated using the GCV (generalized cross validation) method^51^. For all final models, a quasi family with a log link was chosen, enabling the modeling of the dispersion parameter based on the available data. The adequacy of the models was assessed by visually examining residual plots and utilizing the diagnostic information generated using the gam.check function in R^52^.

Model selection procedures adhered to established methods^51,52^, wherein an initially fitted fully saturated model was created for each response variable. The final model was then selected based on evaluation criteria such as the GCV score, percentage of deviance explained, and assessment of fit through examination of residual plots. The simplest model (i.e., model with fewest terms) was chosen based on a decrease in the GCV score and an increase in the explained deviance. Terms were assessed for removal if they met either of the following criteria: (1) linear terms that were statistically non-significant with parameter coefficients close to 0, or (2) smoothed terms that were statistically non-significant with estimated degrees of freedom (edf) close to 0. The linear form of the term was retained if excluding the smoothed term, which had an estimated degrees of freedom (edf) close to 0, resulted in an improvement in the explained deviance without a decrease in the GCV score. Explanatory variables were assessed for multicollinearity, and in cases where it was detected, the term with the least support for inclusion in the final model, as per the model selection criteria, were removed.

For each term in the best fit models, individual variable plots (labelled as A) are provided. The y-axis represents the effect of the covariate on the estimated response, with a value of 0 indicating no effect. Values above 0 indicate a positive relationship, while values below 0 indicate a negative relationship. The x-axis in each variable plot includes small vertical ticks denoting the observation locations, also known as a rugplot. To display the absolute value of the response variable (debris count) as a function of the explanatory variable(s), the *predict* function in the stats package^48^ was used for each of the best fit models and plots created to show the relationship (labelled B).

### Changes in debris accumulation rates before and during COVID-19 restrictions

To facilitate comparison of daily accumulation rates, only surveys conducted during the same months were used, as seasonality is known to impact accumulation rates on Maui’s beaches^11,14,17^. To compare daily accumulation rates at Sites 1 and 2 before and during COVID-19, a one-way ANOVA^53^ was used where daily debris accumulation rates for each site were divided into a pre-and-during COVID-19 category. A subset of debris classified as land sourced^17^ was also investigated as it was the category most likely to be impacted by the COVID-19 restrictions. Details on the division of debris into land-based and non-land-based categories are found in Supplemental Materials Table 2.

## Results

**Figure 3:**
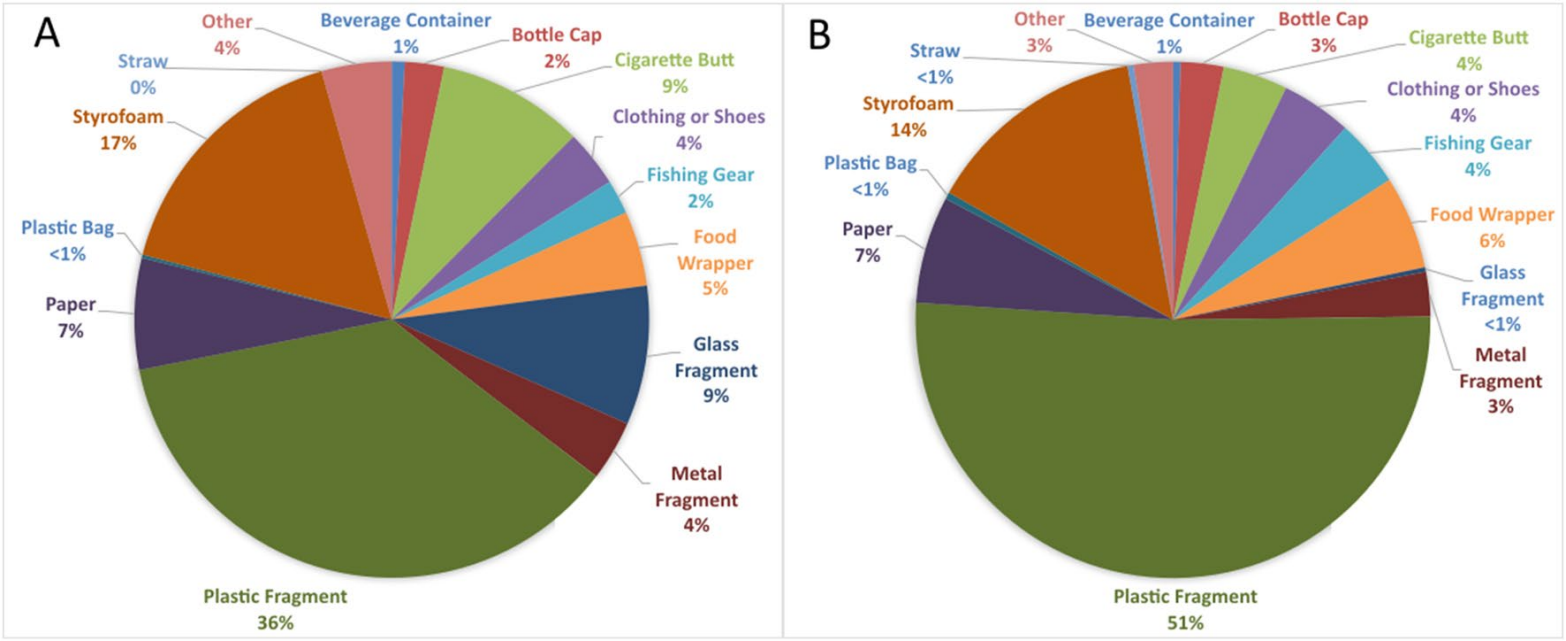
Major debris categories classified at Sites 1 (**A**) and 2 (**B**) over 51 daily accumulation surveys conducted from May 6, 2020 to May 27, 2021.

During the COVID-19 pandemic, data collection efforts resulted in 102 surveys conducted between May 6, 2020 and May 27, 2021, with 51 daily accumulation surveys carried out at each of the sites. The average daily accumulation rates at Sites 1 and 2 during the initial surveys in May 2020 were 2.5 and 15.2 items/100 m/day, respectively, while on the final survey dates in May 2021 when restrictions were eased, they measured 60.2 and 16.5 items/100 m/day, respectively. In total, 978 items were removed from site 1 and 2,084 from Site 2. Plastic fragments constituted the majority of items observed at both Sites 1 and 2 (Fig. 3), with Site 1 displaying an average accumulation rate of 7 plastic items/100 m/day, while Site 2 recorded a higher rate of 20.9 plastic items/100 m/day. Before the COVID-19 pandemic, 42 surveys were conducted, with 21 daily accumulation surveys carried out at each of the sites between July 26, 2017 to December 25, 2017, resulting in the removal of 899 debris items at Site 1 and 388 debris items at Site 2.

**Table 1:** Summary of top GAM models showing the relationship between coastal debris and the COVID-19 Pandemic from May 2020 to May 2021, where rows represent candidate explanatory variables and columns represent response variables.

	Site 1 *Kam'aole Beach*	Site 2 *Ulua Beach*
Intercept	3.57***	3.48***
Julian day	s(5.00)***	s(4.63)***
Lockdown level 1	–	–
Lockdown level 2	0.33	–
Lockdown level 3	− 3.10**	–
Lockdown level 4	− 1.48	–
Lockdown level 5	− 3.84**	–
Relative Exposure Index (REI)	–	s(6.46)
Relative Tidal Range (RTR)	–	s(7.77)
Deviance explained (%)	72.4	79.1
Number of observations	51	48

The parametric coefficient estimates for factors and the degree of smoothing, s(EDF), for smooth terms included in the final model are presented in the cells.

Significance of each model term is indicated by an * where: *P = 0.01–0.05; **P = 0.001–0.01; ***P = 0–0.001; *P* = 0.05–0.1

Cells with a “–” represent terms dropped from the final model.

### Changes in debris accumulation rates throughout the COVID-19 restrictions

Lockdown level and Julian day were found to significantly influence debris accumulation during the first year of the COVID-19 pandemic, after accounting for potential impacts of environmental parameters (Table 1). After model selection, environmental variables were retained for Site 2 only, with varying impacts on coastal accumulation (Supplementary Fig. 1).

**Figure 4:**
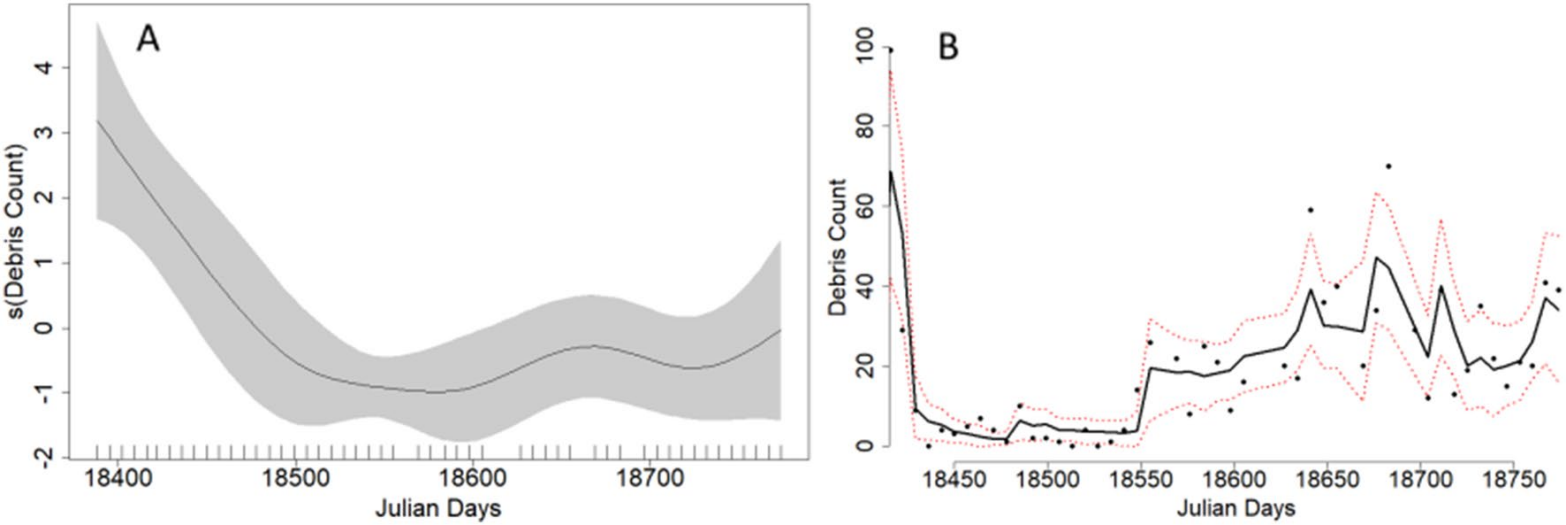
Results from the best fit generalized additive model (GAM) for debris counts at Site 1 (Kam‘aole Beach) showing (**A**) model parameter estimates of Julian Day (where the first day is May 6, 2020 and last day is May 27, 2021) and (**B**) raw debris counts (points) and model predicted debris counts (line) per 100 m/day based on Julian Day. The shaded and dashed lines represent the 95% confidence intervals of the parameter estimates and fitted values respectively. The vertical ticks on image A indicate the day of observations (i.e. a rugplot).

The best fit model for Site 1 explained 72.4% of the deviance, incorporating a smoothed term for date (Julian day) and a categorical term for lockdown level (Table 1), while the best fit model for Site 2 explained 79.1% of the deviance, featuring smoothed terms for date (Julian day), REI, and RTR (Table 1, supplementary materials Fig. 1). Coastal debris accumulation varied significantly throughout the pandemic and was highest at the onset of the pandemic decreasing to a low in October 2020 before increasing again (Table 1; Fig. 4).

**Figure 5:**
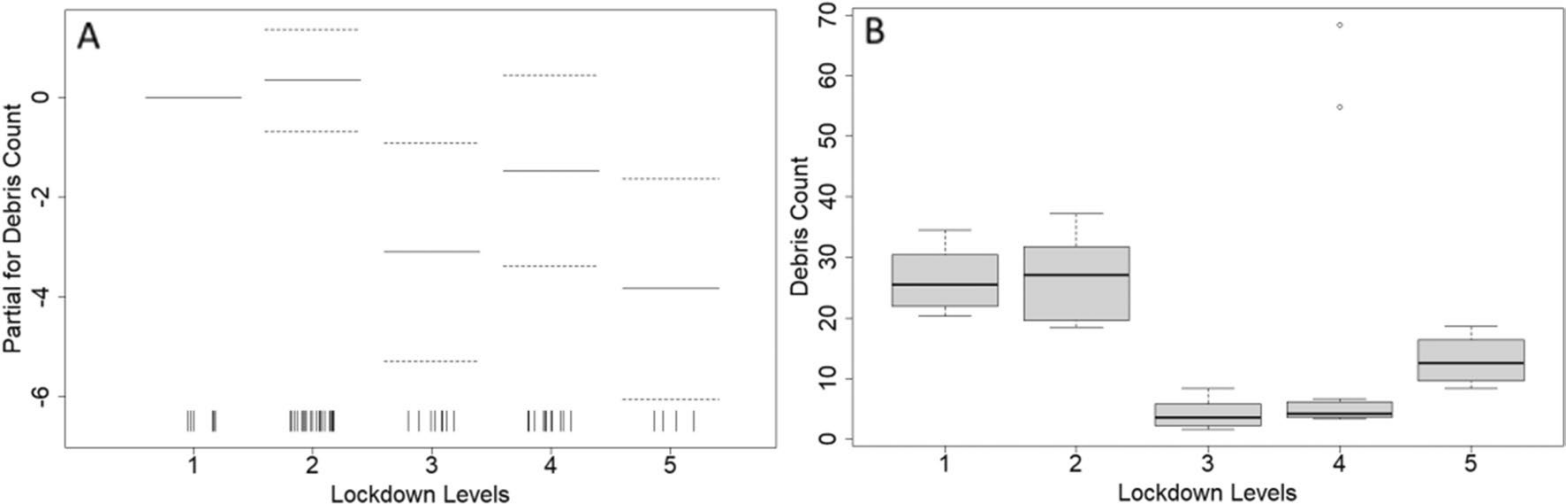
Results from the best fit generalized additive model (GAM) for debris counts at Site 1 (Kam‘aole Beach) showing (**A**) model parameter estimates of lockdown levels (1 = low 5 = high) and (**B**) model predicted debris counts based on lockdown level. Dashed lines in image A represent the 95% confidence intervals of the parameter estimate and vertical ticks indicate the number of observations in each category (i.e. a rugplot).

Lockdown levels significantly impacted coastal debris accumulation at Site 1 (Table 1; Fig. 5) with debris accumulation being lowest during higher lock down levels (i.e. 3, 4, and 5).

**Figure 6:**
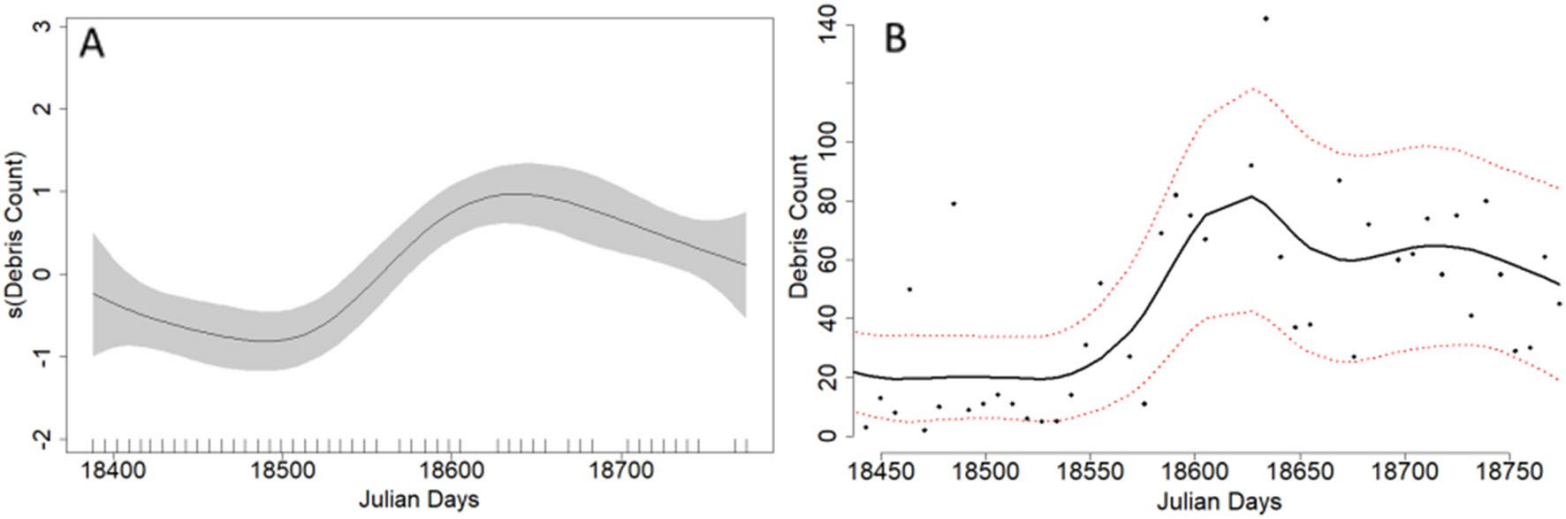
Results from the best fit generalized additive model (GAM) for debris counts at Site 2 (Ulua Beach) showing (**A**) model parameter estimates of Julian Day (where the first day is May 6, 2020 and last day is May 27, 2021) and (**B**) raw debris counts (points) and model predicted debris counts per 100 m/day based on Julian Day. The shaded and dashed lines represent the 95% confidence intervals of the parameter estimates and fitted values respectively. The vertical ticks on image A indicate the day of observations (i.e. a rugplot).

Coastal debris accumulation varied significantly throughout the pandemic and rose significantly after July 2020, remaining high from October 2020 onward at Site 2 (Table 1; Fig. 6).

### Changes in debris accumulation rates before and during COVID-19 restrictions

**Figure 7:**
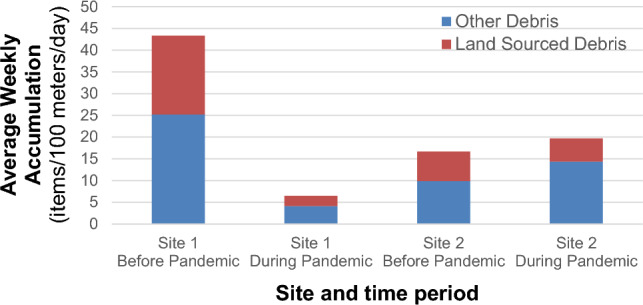
A comparison of average daily debris accumulation rates at Sites 1 and 2 before (2017) and during (2020) the COVID-19 Pandemic, spanning August 26 to November 3.

To focus on the period of stringent lockdown measures and minimal tourism arrivals, we conducted a comparative analysis of accumulation rates specifically for Sites 1 and 2, covering the months of August 26 to November 3 in both 2017 and 2020. Comparison of the average daily accumulation rates during this period found that the anthropause caused significant decreases [F(1,27) = 17.75, p-value < 0.001] in the observed accumulation rates at Site 1, while having no impact [F(1,27) = 0.24, p-value = 0.626] on Site 2 (Fig. 6). Comparison of land-sourced debris accumulation rates during the anthropause at Site 1 found a ~ 85% reduction in debris accumulation rates when compared to the same months in 2017 (Fig. 7).

## Discussion

High debris accumulation remains a problem for the Hawaiian Archipelago^14,17,54^, with both local and remote sources contributing to the issue^55^. Here, we show that lower beach use reduced overall debris load at two beaches, and at one beach resulted in a tenfold decrease of land-based debris. Further, both beach sites showed lower debris accumulation rates prior to easing of travel restrictions from the continental USA, after which accumulation rates rose significantly. By shedding light on the vectors for debris accumulation along Hawaiʻi’s coastline, these findings emphasize the critical need for ongoing monitoring and management of both debris accumulation and general waste pathways, highlighting the significance of proactive measures to protect the natural environment.

The observed fluctuations in debris accumulation at both study sites were likely driven by changes in the number of people arriving on Maui over the course of the COVID-19 pandemic and subsequent beach use. The day prior to declaring a global pandemic, 3,935 passengers arrived on Maui from the continental USA alone^31^. Fifteen days later, when the 14-day quarantine period was instated, this number dropped to 54 passengers^31^. The 14-day mandatory quarantine limited the number of daily visitors to Maui (average daily arrival = 92), which is significantly lower when compared to prior years (2019: average daily arrivals = 6,574 passengers; 2018: average daily arrivals = 5,960 passengers)^31^. Coastal use and population size have been linked to debris accumulation^16,54^, and are likely contributing factors to debris accumulation on beaches observed in this study. As COVID-19 restrictions were eased, debris loads began to rise from July onwards, and reached a peak in October, when the daily passenger arrivals reached over 1,000 individuals^31^. The significance of date and lockdown level on debris accumulation at both sites suggests a strong connection between total population (tourists and residents) and debris loads. Nevertheless, it is essential to acknowledge that continuous monitoring of beach usage was not practical, and the assumption is made that the date is correlated with beach usage, particularly tourists, given that most visitors frequent the beaches during their stay^33^. Moreover, beach debris accumulation is influenced by a myriad of factors, with inputs originating from both coastal land-based areas and offshore coastal waters, the latter of which would not be influenced by COVID-19 restrictions. While these influences are expected to be minor given the small survey area and short duration between debris counts, it is crucial to remain mindful of their potential impact when interpreting the results.

There are many anthropogenic drivers influencing the deposition of land-based debris loads^11,14,17,54^, including the degree of use and type of site, tourism activity, local population size, and waste management systems. In Maui, beach use is impacted significantly by tourism numbers, which fluctuate depending on the time of year; both were reduced significantly during the COVID-19 pandemic. Site 1, Kam‘aole Beach Park III, is one of the most popular beaches on Maui due to its size, accessibility, barbeques and picnic tables for social gatherings, and proximity to shops, restaurants, and numerous accommodation options for tourists. As such, Site 1 is heavily utilized by tourists and received minimal traffic during the initial months of the COVID-19 pandemic, particularly during the highest lockdown levels (levels 3–5). Observed debris trends were also lowest during this time period at Site 1, and decreased steadily as debris was removed and deposition arising from beach use was minimal due to the lockdown. Recent studies looking at debris accumulation trends resulting from COVID-19 showed similar results, with some quantifying a 60–70% reduction in debris accumulation due to beach access restrictions^56^.

At both beaches, increases in debris accumulation trends coincided with reduction in lockdown level, with Site 1 experiencing initial increases at lockdown level 2 or less and Site 2 at a lockdown level 3 or less. In both cases, debris accumulation is likely related to beach use. However, by September/October of 2020, Site 2 accumulation rates were similar to those observed in 2017 during the same months, suggesting a faster return to pre-COVID-19 rates than Site 1. Site 2 may experience more influence from coastal/offshore debris sources that are affected by seasonal variations in wind patterns. Beach features, such as slope, waves, and shape^39^ are also know to impact debris accumulation^14^ and may have also been a factor here. These influences could provide insight into the unexpected increase in debris during COVID restrictions, as they would be minimally impacted by lockdown restrictions. Globally, similar trends of reduced litter and coastal debris were observed during the COVID-19 lockdowns^25,26,56–58^, and highlights humans as a key vector for debris transport and deposition into coastal environments.

The link between tourism and beach debris has been made in other regions and attributed to a vacation mindset that results in poor waste management practices^15^, aligning with the results reported here. However, on average, only ~ 35% of observed debris items in Maui can be traced back to land-based sources^17^, highlighting a larger issue for beach debris accumulation. Marine debris can enter the ocean environment through a variety of sources, but in most cases represents a misuse or mishandling by an individual or organization^1^. As such, effective marine debris mitigation is only possible when tackled with effective legislation and education. A myriad of narrowly focused policies have been passed by the state of Hawaiʻi^59^ to address waste prevention, however, the majority of the focus in Maui County and the State of Hawai ‘i is on waste management^60^ rather than waste prevention.

The isolated nature of the Hawaiian Islands, coupled with a strong tourism industry, make source reduction a critical step towards realizing reduction of debris in coastal environments. Indeed, the United States Environmental Protection Agency lists source reduction as the highest priority in managing waste, with treatment and disposal the lowest priority^61^, highlighting the backwards approach currently applied in Hawaiʻi and the need for a more effective approach. Reducing waste by limiting the type and amount of material that can be produced in or shipped to Hawaiʻi and developing policies that prevent the transport of products and packaging known to impact coastal environments is a critical next step. A growing body of literature underlines the type of debris items that pose a problem to the ocean^12,17,34,62^ and the impacts they have on birds^63–65^ cetaceans^2,66^ and other marine life^2,67^ in Hawaiʻi. Here, we find plastics to again be a major contributor to beach debris. Although additional research is needed in some areas to quantify the impact of marine debris on coastal environments, there is sufficient data to support (1) education campaigns that target tourists and residents alike and promote responsible waste management practices; (2) a shift in the focus of waste management policies to prioritize waste prevention. By addressing the vacation mindset that contributes to poor waste management practices and developing legislation that encourages waste prevention, we can begin to reduce the amount of debris that ends up in the coastal environments and impacts marine life of all sizes.

### Supplementary Information

Supplementary Information.

## Data Availability

The data used in this study is available upon request. Please contact jenscurrie@pacificwhale.org for access to the data.
