# Supplementary material for: The impact of the anthropause caused by the COVID-19 pandemic on beach debris accumulation in Maui, Hawaiʻi
Source: Sci Rep. 2023 Oct 18;13:17729. doi: 10.1038/s41598-023-44944-4 (PMC10584821; doi:10.1038/s41598-023-44944-4)
Supplement: Supplementary file 1 — Supplementary Information. [file 41598_2023_44944_MOESM1_ESM.pdf]

The impact of the anthropause caused by the COVID-19 pandemic on beach debris accumulation in Maui, Hawai‘i

Jens J. Currie*[1], Florence A. Sullivan[1], Elizabeth Beato[1], Abigail F. Machernis[1], Grace L. Olson[1], Stephanie H. Stack[1,2]

[1]Pacific Whale Foundation, Wailuku, HI 96793, USA

[2]Pacific Whale Foundation Australia, Urangan, QLD 4655, Australia

Supplementary Materials

Table 1. Categories of restrictions and descriptions used to determine lockdown level.

	Covid-19 Regulation Lockdown Levels				
Category	Level 1	Level 2	Level 3	Level 4	Level 5
Beach Access	Beach Parks Open for Recreation	Beach Parks Open for Recreation	Beach Parks Open for Recreation	Beach Parks Open for Recreation	Beach Parks closed (1) or only open for Essential Activities (2)
Mainland Travel	Mainland Travelers can bypass quarantine with a Pre-test	Mainland Travelers can bypass quarantine with a Pre-test	Mainland Travel Mandatory Quarantine	Mainland Travel Mandatory Quarantine	Mainland Travel Mandatory Quarantine
Interisland Travel	Interisland Travelers can bypass quarantine with a Pretest OR proof of vaccination within state of HI	Interisland Travelers can bypass quarantine with a Pretest (5) *beginning on 11/9/2020	Interisland Travel Permitted without Mandatory Quarantine or Pre-test	Interisland Mandatory Quarantine	Interisland Mandatory Quarantine
Mask Mandates	Mask Mandates; Mask mandates change to only indoor public places 5/25/2021	Mask Mandates (4, 6)	Mask Mandates	Mask Mandates (3)	Face coverings required in public areas except while exercising (3)
Restaurant Industry	Restaurants open for dine-in service	Restaurants open for dine-in service	Restaurants open for dine-in service	Restaurants open for dine-in service	Restaurants are take-out only
Start Date	4/19/2021	10/15/2020	6/16/2020	6/1/2020; 8/6/2020	5/4/2020
End Date	5/31/2021	4/18/2021	8/5/2020	6/15/2020; 10/14/2020	5/31/2021

** (1) Beach Park closures indicate parking lot gates and restroom facilities remain closed and no county lifeguards are on duty. The public can still access the shoreline and ocean for essential activities; (2) Essential activities are defined as "engaging in outdoor activity, such as walking, hiking, running, ocean sports (surfing, stand-up paddle boarding, or fishing); (3) All persons over the age of 5 years old shall wear a face mask or*

cloth covering the nose and mouth while in public settings. This requirement shall not apply to persons engaging in permissible exercise activities, so long as physical distancing requirements are maintained; (4) When persons are stationary and adequately separated, such as occupying a pool lounge chair, beach chair, or towel, no mask is required; however, face coverings must be worn while walking to and from the pool or beach area; (5) Interisland travelers age five and older who provide written confirmation of a negative test result from an approved COVID-19 test administered to the traveler within 72 hours from the last leg of departure prior to arrival into Maui County, are exempt from quarantine; (6) Permitted masks or facial coverings must: completely cover the nose and mouth, have two or more layers of washable, breathable fabric, fit snugly against the sides of your face without gaps. Unpermitted masks or face coverings include masks with exposed external valves, bandanas, face shields without a permitted mask underneath.

Table 2. Indicator debris items classified as land sourced.

Ocean-based	Land-based	Other-items
Nylon rope/net fragments	Cigarette filters/cigars	Beverage bottles
Buoys/floats	Straws	Plastic bags
Fishing lures/line	Balloons	Packing straps
Spools	Fireworks	Bottle/container caps
	Golf balls	Other jugs/containers
	Golf tees	
	Syringes	
	Personal care products	
	Flip-flops/slippers	

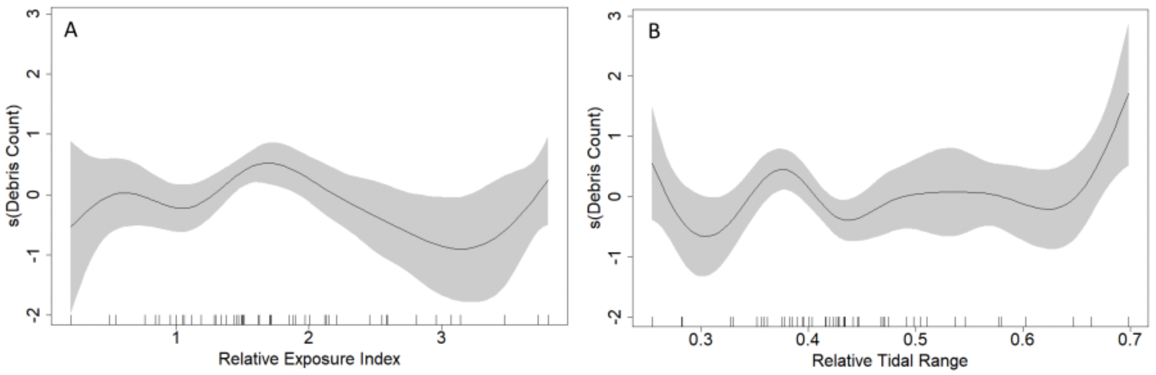

Supplementary Figure 1. Results from the best fit generalized additive model (GAM) for debris counts at Site 2 (Ulua Beach) showing (A) model parameter estimates of Relative Exposure Index and (B) Relative Tidal Range. The shaded lines represent the 95% confidence intervals of the parameter estimates. The vertical ticks indicate the locations of observations (i.e. a rugplot).
